# Visible light guided manipulation of liquid wettability on photoresponsive surfaces

**DOI:** 10.1038/ncomms14968

**Published:** 2017-04-25

**Authors:** Gibum Kwon, Divya Panchanathan, Seyed Reza Mahmoudi, Mohammed A. Gondal, Gareth H. McKinley, Kripa K. Varanasi

**Affiliations:** 1 Department of Mechanical Engineering, Massachusetts Institute of Technology, Cambridge, Massachusetts 02139, USA; 2 Physics Department, King Fahd University of Petroleum and Minerals, Dhahran 34464, Saudi Arabia

## Abstract

Photoresponsive titania surfaces are of great interest due to their unique wettability change upon ultraviolet light illumination. However, their applications are often limited either by the inability to respond to visible light or the need for special treatment to recover the original wettability. Sensitizing TiO_2_ surfaces with visible light-absorbing materials has been utilized in photovoltaic applications. Here we demonstrate that a dye-sensitized TiO_2_ surface can selectively change the wettability towards contacting liquids upon visible light illumination due to a photo-induced voltage across the liquid and the underlying surface. The photo-induced wettability change of our surfaces enables external manipulation of liquid droplet motion upon illumination. We show demulsification of surfactant-stabilized brine-in-oil emulsions via coalescence of brine droplets on our dye-sensitized TiO_2_ surface upon visible light illumination. We anticipate that our surfaces will have a wide range of applications including microfluidic devices with customizable wettability, solar-driven oil–water clean-up and demulsification technologies.

## Abstract (web summary)

Controlling surface wettability using visible light is highly attractive for a range of liquid separation technologies. Here, Varanasi, McKinley and colleagues fabricate dye-sensitized photocatalytic TiO_2_ surfaces on which liquid droplet motion can be externally manipulated by visible light illumination.

Photo-responsive surfaces are of great interest due to the wettability change induced upon exposure to incident light^1^,^2^,^3^. One way to achieve photo-induced wettability change is utilizing photochromic molecules that display reversible transformations upon illumination between two chemical states having different dipole moments or polar moieties^4^,^5^,^6^. However, such organic photochromic molecules typically exhibit low contact angle change upon contact with liquid droplets^4^,^6^,^7^,^8^. Recently, it has been reported that photo-generated charge carriers (electron–hole pairs) can change the capacitance of semiconducting silicon dioxide which in turn change the wettability toward a contacting liquid^9^,^10^. The wettability change is amplified when the semiconductor is subject to an externally applied voltage along with light illumination^9^,^10^.

Photo-responsive titania (TiO_2_) surfaces have demonstrated wettability change upon exposure to ultraviolet light^1^,^3^. A great deal of work has been devoted to elucidating the origin of the well-documented unique ultraviolet light-induced wettability change on TiO_2_ surfaces^2^,^11^,^12^,^13^. Although this continues to remain an active area of research, it is widely accepted that photo-generated electrons and holes change the surface chemistry so that it is favourable for contacting liquids to spread either by photocatalytic oxidation of surface adsorbed organic species^11^,^12^ or by the increase of hydroxyl species due to dissociative water adsorption^2^,^13^.

Photo-driven manipulation of liquid motion on a TiO_2_ surface is highly attractive because it would eliminate any need for either direct electrical contact with liquids or complex electronic circuitry^14^,^15^. However, their practical applications are often limited either by the inability to respond to the visible light spectrum of natural sunlight^16^,^17^ or by the slow kinetics and the need for special environments (that is, storage in dark or heat) to recover the original wetting state^18^,^19^.

The strategy of sensitizing TiO_2_ surfaces with dopants and visible light-absorbing materials (for example, organic dyes) has been utilized in photovoltaics to efficiently absorb solar radiation and convert it to electrical energy^20^,^21^. Imbibition of a liquid dye or adsorption of dye to a TiO_2_ surface is an alternative approach for generating charge carriers upon light illumination^20^,^21^,^22^. On a dye-sensitized TiO_2_ surface, the optical absorption and charge-generating functions are achieved by excitation of dye and subsequent injection of charge carriers (for example, electrons) into the conduction band of TiO_2_ (refs 22, 23, 24). The light absorption behaviour of a dye-sensitized TiO_2_ surface can be readily tuned by careful consideration of the energy levels of the selected dye. Ruthenium (II) polypyridyl complexes have received particular interest due to their wide absorption range from the visible to the near-infrared regime and high stability in the oxidized state^25^. Furthermore, introduction of a textured roughness or porosity to the TiO_2_ surface can dramatically increase the light absorption efficiency due to an increased specific surface area providing capillary stabilization of a surface adsorbed liquid film in which a large number of dye molecules can be directly adsorbed^20^,^21^. Consequently, dye-sensitization may provide a versatile tool to tune the wettability of TiO_2_ surfaces under visible light illumination.

In this work, we demonstrate that a dye-sensitized TiO_2_ surface—fabricated using straightforward dip-coating method—can be engineered to have its wettability state optically modulated upon illumination by visible light. We show that this wettability change arises due to the electric potential difference established between the surface and the liquid upon incident illumination. A systematic study of the relationship between the energy levels of the dye and the contacting liquid reveals that the highest occupied molecular orbital (HOMO) energy level of the dye and the reduction potential of the liquid govern the ensuing wetting behaviours. Utilizing this photo-induced wetting of our dye-sensitized TiO_2_ surface we demonstrate light-guided manipulation of liquid droplet motion along the surface. Furthermore, we show demulsification of surfactant-stabilized brine-in-oil emulsion via interfacial coalescence of brine droplets under visible light illumination. Such surfaces thus offer a wide range of potential applications including optically driven, microfluidic devices with customizable wettability^5^,^26^ and continuous solar-driven self-cleaning and oil–water separation technologies^27^,^28^.

## Results

### Wetting behaviours of liquids on an N3 dye-sensitized TiO_2_

**Figure 1:**
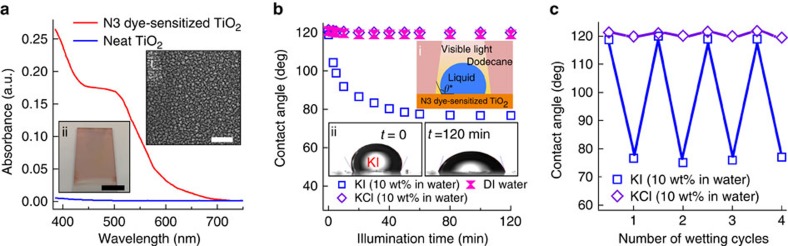
Wetting behaviours of liquids on an N3 dye-sensitized TiO_2_ surface. (**a**) Visible light absorption data of an N3-sensitized TiO_2_ surface. Corresponding absorption data for an unsensitized neat TiO_2_ surface is also shown for comparison. Insets: (i) a scanning electron microscopic (SEM) image of a nanostructured TiO_2_ surface. Scale bar, 200 nm. (ii) A photograph of an N3 dye-sensitized TiO_2_ surface. Scale bar, 1 cm. (**b**) Evolution in the measured equilibrium contact angles for potassium iodide (KI, 10 wt% in water), deionized (DI) water and potassium chloride (KCl, 10 wt% in water) on an N3 dye-sensitized TiO_2_ surface as a function of illumination time (intensity=145 mW cm^−2^). Insets: (i) a schematic of *in situ* contact angle measurement of an aqueous droplet on a dye-sensitized TiO_2_ surface submerged in dodecane under visible light illumination. (ii) Photographs of a KI droplet placed on an N3 dye-sensitized TiO_2_ surface submerged in dodecane before (*t*=0) and after visible light illumination (*t*=120 min). (**c**) A plot of evolution in contact angles for multiple wetting cycles of KI and KCl droplets on an N3 dye-sensitized TiO_2_ surface under visible light illumination.

We fabricated an N3 dye-sensitized TiO_2_ surface. A thin, nanostructured and highly porous TiO_2_ surface is prepared by layer-by-layer (LBL) deposition of negatively charged TiO_2_ nanoparticles (average diameter ≈20 nm) and positively charged poly(allylamine hydrochloride) (PAH) on an indium tin oxide (ITO)-coated glass slide followed by calcination (see Methods). A scanning electron microscopy image (see inset (i) in Fig. 1a) shows that the surface is highly porous with a large specific surface area. The ratio of the total surface area per unit projected area is estimated to be 56 (see Supplementary Note 1). The resulting porous TiO_2_ surface is subsequently dip-coated in N3 dye solution. N3 dye molecules chemisorb to the surface via carboxylate groups^29^,^30^. Adsorption of N3 dye molecules leads to a deep brown coloration of the surface (see inset (ii) in Fig. 1a). We measured the visible light absorption spectra of an N3 dye-sensitized TiO_2_ surface and, as shown in Fig. 1a, our surface absorbs a broad range of the incident visible spectrum (390 nm≤*λ*≤700 nm). In contrast, an unsensitized LBL-deposited TiO_2_ nanostructured surface displays negligible absorption in the visible regime due to the wide band gap energy of the TiO_2_ particles (≈3.2 eV)^31^.

To study the photo-induced wettability change of our N3 dye-sensitized TiO_2_ surface, we measured the evolution in the contact angles *in situ* for three liquid droplets: deionized (DI) water, potassium iodide (KI, 10 wt% in water) and potassium chloride (KCl, 10 wt% in water). *In situ* contact angle measurements were conducted under oil (for example, dodecane) environment to minimize evaporation of a droplet due to the heat generated from the light source (see inset (i) in Fig. 1b and Methods) and to simulate the condition expected in oil–water separation operation^32^,^33^,^34^,^35^. The visible light source with intensity (*I*=145 mW cm^−2^) is projected from above the surface to minimize intensity loss along the light transmission path. Figure 1b shows the evolution in the macroscopic contact angles (θ*) for DI water, KI and KCl droplets as a function of illumination time. The equilibrium contact angles for DI water and KCl remain almost constant during illumination (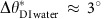 and 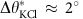 where 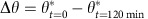), while those for KI decrease progressively from 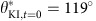 with increasing illumination time before finally approaching 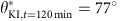 (see Fig. 1b). After it reaches 77°, the contact angle remains unchanged. Similar contact angle saturation is also typically observed in conventional electrowetting-on-dielectric applications^36^. Very similar contact angle changes were also observed on a thicker N3 dye-sensitized TiO_2_ film prepared with 45 bilayers of TiO_2_ and PAH. We also observed that the negligible wettability change for KCl was independent of concentration (see Supplementary Table 1) while KI droplets with higher ionic concentration exhibited rapid decrease in contact angles (see Supplementary Fig. 1 and Supplementary Note 2). Note that unlike the conventional electrowetting (or photoelectrowetting) where the contact angle change is effected within milliseconds upon (high) voltage application^37^, our N3 dye-sensitized TiO_2_ surface exhibits a wettability change for KI droplet over tens of minutes. However, the switchability does not require application of large voltages, and can be effected using droplets on surfaces that are immersed in alkanes or other low surface tension organic liquids. The expected application areas will thus be very different to conventional electrowetting devices. Our selective photo-induced wetting response of KI over KCl (or DI water) is further exemplified by considering multiple wetting cycles as shown in Fig. 1c. The contact angles for fresh droplets of KI placed at a fixed location on the surface cycles between 
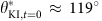
 and 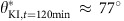 under repeated illumination, while that for KCl remains almost constant. Unlike an unsensitized, as-fabricated TiO_2_ surface, X-ray photoelectron spectroscopy analysis of our N3 dye-sensitized TiO_2_ surface clearly indicates that the surface chemistry remains unaffected after multiple cycles of wetting under visible light illumination (see Supplementary Fig. 2 and Supplementary Note 3).

### The origin of the photo-induced wettability change

On dye-sensitized TiO_2_ surfaces, photo-generated electrons from incident visible light illumination are injected and travel through the nanoporous TiO_2_ network^23^,^24^. We can anticipate that this electron transfer results in the generation of an electric potential difference between the surface and the contacting liquid that can then induce electrowetting effects in liquid droplets on the surface. To understand this further, we first consider the physicochemical origin of a photo-induced electric potential difference under light illumination.

**Figure 2:**
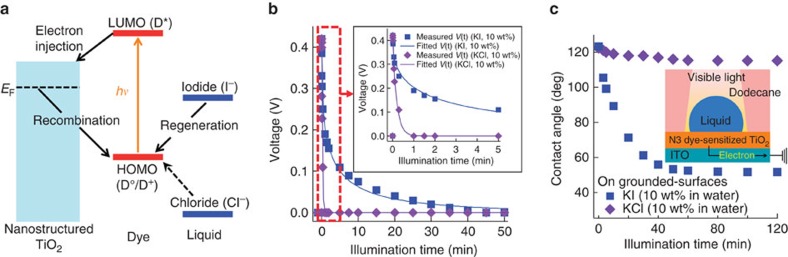
The origin of the photo-induced wettability change of an N3 dye-sensitized TiO_2_ surface. (**a**) Schematic illustration and relative energy state diagram of an N3 dye-sensitized TiO_2_ surface contacting either KI or KCl droplet. (**b**) A plot of measured voltages across the contacting liquids (KI or KCl) and the N3 dye-sensitized TiO_2_ surface under visible light illumination (intensity=145 mW cm^−2^). Inset: a zoomed-in image showing voltages immediately after the onset of visible light illumination. (**c**) Evolution in the measured contact angles for KI and KCl droplets on the electrically grounded N3 dye-sensitized TiO_2_ surface. Inset: a schematic illustration of *in situ* contact angle measurement of a droplet on the electrically grounded dye-sensitized TiO_2_ surface.

Figure 2a shows a schematic and energy diagram of an N3 dye-sensitized TiO_2_ surface contacting a KI droplet. After electron transfer upon illumination, a region over which the charge distribution differs from the bulk is produced. This corresponds to the electrolytic double layer and the accumulation layer at the contacting KI and TiO_2_, respectively^22^. Consequently, an electric potential difference (that is, a measurable voltage) is generated between the contacting KI droplet and the underlying substrate. The oxidized dye (D^+^) can be subsequently reduced by accepting an electron from the reducing agent (that is, the iodide, I^−^) in the KI through the chemical reaction (a process known as regeneration)^38^,^39^. An alternative pathway for reducing the oxidized dye is by recombining with an electron in the TiO_2_ (that is, the process of recombination)^40^,^41^. While the regeneration is more favourable and orders of magnitude faster^42^,^43^, the recombination also plays an important role in dye reduction process, especially under open-circuit conditions^44^,^45^.

To validate our hypothesis, we measured the *in situ* voltage across the contacting liquids and the N3 dye-sensitized TiO_2_ surface while illuminating with visible light (see Fig. 2b). Immediately after the onset of illumination, a potential difference *V*_KI, *t=*0_ ≈0.42 V is observed between the KI and the lower ITO electrode. The voltage decreases gradually with increasing illumination time and eventually reaches zero after *t*≈45 min. Similar to photoelectrowetting^9^,^46^ in which the voltage along with optically generated charge carriers leads to wetting, the photo-induced voltage causes spreading of the KI droplet on our N3 dye-sensitized TiO_2_ surface. The gradual decrease in the measured voltage can be explained by considering electrolytic double layer as a capacitor (C) and the underlying TiO_2_ film as a resistor (R). Thus, the KI droplet and the TiO_2_ film can be considered as an RC circuit connected in series. When the capacitor in an RC circuit is discharged, the voltage *V*(*t*) is given by


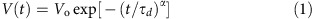


where *V*_o_, *τ*_d_ and *α* denote the voltage at *t*=0, the relaxation time constant and the fractional derivative order, respectively. Note that equation (1) characterizes a fractional RC circuit in which the capacitor (or a resistor) is imperfect^47^. For example, the electrolytic double layer here may be a leaky capacitor due to the defects present on the surface of the nanoparticles. Our measured voltages across the KI and ITO (see Fig. 2b) match well with equation (1) with *τ*_d_=130 s and *α*=0.42 (see Supplementary Note 4). Furthermore, we observed that changes in the contact angle halt instantaneously and the droplet shape remains unchanged when the illumination is turned off (see Supplementary Fig. 3 and Supplementary Note 5).

Unlike the gradual decrease in voltage between the KI droplet and the surface during optical illumination, we observed a rapid decrease in the voltage between a KCl droplet and the surface after the onset of illumination (see Fig. 2b). Rapid decrease in the voltage highlights the importance of the dye reduction process in photo-induced voltage generation. As the reduction potential of iodide (I^−^, *E*_red, I_−=0.53 V versus normal hydrogen electrode (NHE))^48^ is above (that is, less positive than) the HOMO energy level of N3 dye (*E*_HOMO, N3_=1.0 V versus NHE)^49^, it presents a driving force to reduce the oxidized dye. As a consequence, an N3 dye-sensitized TiO_2_ surface contacting a KI droplet can maintain a prolonged voltage difference by suppressing the recombination process. In contrast, chloride (Cl^−^, *E*_red, Cl_−=1.36 V versus NHE)^48^ cannot effectively reduce the oxidized N3 dye due to its higher reduction potential resulting in a dominant recombination process.

To probe our hypothesis that suppressing the recombination process leads to prolonged photo-induced voltage generation, and subsequently extension of the wetting (that is, a larger change in contact angles), we fabricated an electrically grounded N3 dye-sensitized TiO_2_ surface (see inset in Fig. 2c). In contrast to the previous surface where photo-generated electrons continue to accumulate and participate in the recombination process, the electrically grounded surface minimizes electron accumulation. Figure 2c shows an enhanced decrease in macroscopic contact angles for KI 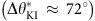 and KCl 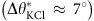 droplets on the electrically grounded N3 dye-sensitized TiO_2_ surface after 120 min of illumination. We anticipate that this is a direct consequence of the suppression of the recombination process.

### Photo-induced wetting on various dyes-sensitized TiO_2_

**Figure 3:**
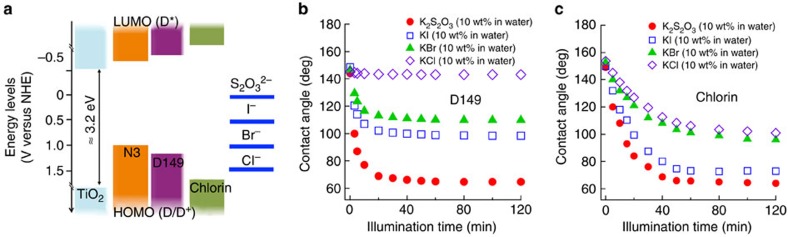
Photo-induced wetting of contacting liquids on various dye-sensitized TiO_2_ surfaces. (**a**) An energy diagram of various dyes (N3, D149 and Chlorin) and 10 wt% ionic aqueous solutions (potassium thiosulfate (K_2_S_2_O_3_), KI, potassium bromide (KBr) and KCl). The HOMO energy levels of the dyes are located in between the reduction potential of liquids considered in this work. (**b**,**c**) Evolution in the measured contact angles for 10 wt% ionic aqueous K_2_S_2_O_3_, KI, KBr and KCl droplets on a D149 dye-sensitized TiO_2_ surface and on a Chlorin dye-sensitized TiO_2_ surface submerged in dodecane, respectively, as a function of illumination time.

These findings described above provide us with design parameters to systematically manipulate the wettability of dye-sensitized TiO_2_ surfaces towards different contacting liquids in response to visible light illumination. For effective wetting of specific liquids, it is preferential to regenerate the oxidized dye by a reducing agent within the contacting liquid (see Fig. 2a). This often requires careful consideration of the energy levels of the dye and different contacting liquids. As is well documented in the dye-sensitized solar cell literature^23^,^24^, effective dye regeneration is typically achieved by using electrolytes that possess a reduction potential that is less positive than the HOMO energy level of dye. This enables us to create a design chart for photo-induced wetting of contacting liquids on a dye-sensitized TiO_2_ surface. Figure 3a shows an energy diagram of various dyes and electrolytes. Here we chose D149 dye^50^ and Chlorin dye^51^ as sensitizers (see Methods). Similar to our N3 dye-sensitized TiO_2_ surface, a D149 dye-sensitized TiO_2_ and a Chlorin dye-sensitized TiO_2_ absorb across a broad range of the visible light spectrum (see Supplementary Fig. 4 and Supplementary Note 6). As their lowest unoccupied molecular orbital (LUMO) energy levels are above (that is, less positive than) the conduction band of TiO_2_, efficient electron transfer can be achieved^50^,^51^,^52^. However, subsequent regeneration of the oxidized dyes by various electrolytes will be selective due to their different HOMO energy levels (*E*_HOMO, D149_=1.14 V versus NHE^50^ and E_HOMO, Chlorin_=1.72 V versus NHE^51^) with respect to the reduction potentials of various electrolytes. Along with KI and KCl, we utilized potassium thiosulfate (K_2_S_2_O_3_, 10 wt% in water, *E*_red,_ K_2_S_2_O_3_=0.08 V versus NHE)^48^ and potassium bromide (KBr, 10 wt% in water, *E*_red, KBr_=1.09 V versus NHE)^48^ as contacting liquids. *In situ* photo-induced voltage measurements of a D149 dye-sensitized TiO_2_ and a Chlorin dye-sensitized TiO_2_ in contact with droplets of various electrolytes clearly indicate that a prolonged voltage difference across the surface and the contacting liquid droplet is achieved when *E*_HOMO, dye_>*E*_red, liquid_ (see Supplementary Fig. 5 and Supplementary Note 7). Supplementary Table 2 lists the values of *τ*_d_ and *α* found in the voltage predictions using our RC circuit model. Figure 3b shows the evolution in the macroscopic contact angles for K_2_S_2_O_3_, KI, KBr and KCl droplets on a D149 dye-sensitized TiO_2_ surface under illumination. Contacting liquids with a lower reduction potential (that is, less positive) than *E*_HOMO, D149_ (for example, K_2_S_2_O_3_, KI and KBr, see also Fig. 3a) spread on the surface, while a droplet of KCl exhibits a negligible contact angle decrease. In contrast, we observed a decrease in the macroscopic contact angles for all contacting liquids on a Chlorin dye-sensitized TiO_2_ surface under visible light illumination (see Fig. 3c). This is a direct consequence of the effective dye-regeneration process with all contacting liquids as a result of the very high *E*_HOMO_ of Chlorin dye^51^,^52^. Selective wetting behaviours of K_2_S_2_O_3_ (or KI) over KBr (or KCl) on an N3 dye-sensitized TiO_2_ surface is also observed (see Supplementary Fig. 6 and Supplementary Note 8). To the best of our knowledge, this is the first-ever systematic demonstration of visible light-induced wetting of contacting liquid droplet on dye-sensitized TiO_2_ surfaces.

### Manipulating liquid droplet on a Chlorin dye-sensitized TiO_2_

**Figure 4:**
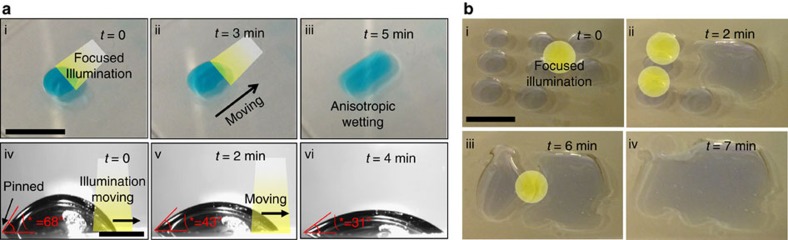
Manipulating droplet motion on a Chlorin dye-sensitized TiO_2_ surface. (**a** (i)–(iii)) A sequence of images showing visible light guided movement of a droplet of sodium chloride (NaCl, 10 wt% in water) on a patterned Chlorin dye-sensitized TiO_2_ surface. Scale bar, 1 cm. ((iv)–(vi)) Sequential images captured from contact angle goniometry of anisotropic wetting of an NaCl droplet (9 μl) upon focused visible light illumination. Scale bar, 2 mm. (**b**) Sequential images of photo-induced coalescence of multiple NaCl droplets placed on a Chlorin dye-sensitized TiO_2_ surface submerged in dodecane. Scale bar, 1 cm.

Our ability to photo-induce the selective wetting of contacting liquids on a dye-sensitized TiO_2_ surface provides a versatile tool to manipulate liquid motion. Figure 4a shows visible light guided movement of a sodium chloride (NaCl; 10 wt% in water) droplet on a patterned dye-sensitized TiO_2_ surface submerged in dodecane. Our patterned surface consists of a thin channel of Chlorin dye-sensitized LBL-assembled nanoporous TiO_2_ surrounded by a hydrophobic background (see Methods). A droplet of NaCl is placed on the channel, and white light is focused on one edge of the droplet (see Fig. 4a (i)). The contact angle at the interface between the liquid and the illuminated surface decreases while the other side remains pinned. Consequently, the droplet's centre of mass moves towards the illuminated edge (see Fig. 4a (ii)). Further illumination leads to an elongation of the droplet shape (see Fig. 4a (iii)). Supplementary Movie 1 demonstrates the photo-induced anisotropic wetting (that is, progressive spreading in one direction while the other side remains pinned) of a NaCl droplet on a Chlorin dye-sensitized TiO_2_ surface. Sequential snapshot images are also shown in Fig. 4a (iv)–(vi). This anisotropic wetting behaviour under focused optical illumination allows for coalescence of multiple aqueous droplets that are initially pinned on the surface. Figure 4b shows sequential photographs of the coalescence of multiple NaCl droplets on a Chlorin dye-sensitized TiO_2_ surface submerged in dodecane. By shining visible light at various locations between the droplets we can photo-induce coalescence of all droplets resulting in a single continuous NaCl aqueous layer on the surface.

### Demulsification of brine-in-oil emulsions

**Figure 5:**
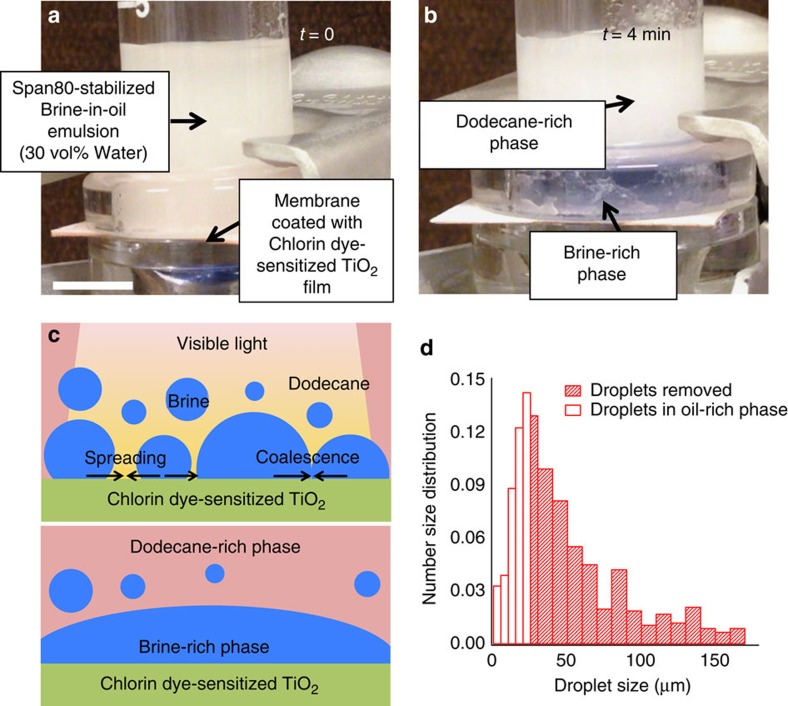
Demulsification of brine-in-oil emulsion. (**a**) A demulsification apparatus with a 30:70 v-v Span80 (0.1 wt%)-stabilized brine (10 wt% NaCl in water)-in-dodecane emulsion above the membrane coated with Chlorin dye-sensitized TiO_2_ film. Scale bar, 2 cm. (**b**) Brine droplets contacting the membrane surface coalesce upon visible light illumination resulting in spontaneous demulsification and gravity separation. (**c**) Schematic illustration of demulsification of brine–oil emulsion via interfacial coalescence of brine droplets on Chlorin dye-sensitized TiO_2_ surface upon visible light illumination. (**d**) Measured number size distribution of brine droplets for the feed emulsion. The shaded region represents the brine droplets removed (corresponding to >99.9 vol%) during demulsification.

This photo-induced coalescence process is an ideal candidate to substitute for conventional electrostatic coalescence technique employed in demulsification^32^,^33^,^34^ (that is, the conversion of an oil–water emulsion into two separate oil-free and water-free phases), especially for mixtures with high salt concentration (for example, brine–oil emulsions) that are typically generated in enhanced oil-recovery operations. The demulsification of brine–oil emulsions using our photo-induced coalescence is highly desirable because conductive emulsions resulting from ionized salts create a current path upon application of an external electric field that hinders generation of induced dipole moments^53^. Here we demonstrate demulsification of a brine–oil emulsion utilizing photo-induced coalescence of water droplets containing salts on a dye-sensitized TiO_2_ surface. Figure 5a,b show demulsification of a brine (10 wt% NaCl in water)-in-dodecane emulsion stabilized by 0.1 wt% of Span80 surfactant (see Methods). The demulsification apparatus consists of a stainless steel membrane (pore size ≈2 μm) coated with Chlorin dye-sensitized TiO_2_ nanostructured film sandwiched between the two vertical glass tubes (see Fig. 5a). The emulsion is added to the upper tube and immediately visible light is illuminated on the membrane surface. Within minutes of illumination, the brine-in-dodecane emulsion demulsifies into brine-rich and dodecane-rich phases (see Fig. 5b). Similar to photo-induced coalescence of NaCl droplets (see Fig. 4b), brine droplets that contact the Chlorin dye-sensitized TiO_2_ mesh surface spread and coalesce with other droplets under white light illumination. Figure 5c shows a schematic illustrating our photo-induced demulsification of brine–oil emulsion. Figure 5d shows the brine droplet number size distribution of the dodecane-rich retentate after 4 min of demulsification. By comparing with the feed emulsion, it is evident that nearly all brine droplets above 25 μm (that is, >99.9 vol% of brine) are removed by photo-induced coalescence. Supplementary Movie 2 demonstrates the demulsification of a brine-in-oil emulsion using photo-induced coalescence of brine droplets on Chlorin dye-sensitized TiO_2_ mesh surface.

In summary, we have demonstrated that dye-sensitized nanoporous TiO_2_ surfaces can selectively change the wettability towards contacting liquids upon visible light illumination due to a photo-induced voltage difference across the liquid and the surface. We have also shown that the HOMO energy level of the selected dye and the reduction potential of the contacting liquid droplet phase govern the effective dynamics of the photo-induced voltage difference. The photo-induced wettability change of a dye-sensitized TiO_2_ surface enables external manipulation of liquid droplet motion across a surface upon visible light illumination. We also demonstrated spontaneous demulsification and separation of surfactant-stabilized brine-in-oil emulsion using photo-induced coalescence of brine droplets on a dye-sensitized TiO_2_ surface. We anticipate that such abilities to remotely activate and control the wettability states of surfaces through optical illumination will enable new microfluidic separation technologies as well as result in new sunlight-driven oil–water clean-up and demulsification approaches.

## Methods

### Fabrication of thin nanostructured TiO_2_ surfaces

A thin, nanostructured TiO_2_ surface was fabricated using LBL deposition either on an ITO-coated glass slide (Sigma Aldrich, surface resistivity=8–12 Ω sq^−1^) or stainless steel mesh (TWP Inc., pore size ≈2 μm) substrates. First, the substrates were thoroughly rinsed with isopropyl alcohol and DI water followed by drying with nitrogen gas. The cleaned substrates were sequentially dip-coated in PAH (Sigma Aldrich, average molecular weight=15,000 g mol^−1^) aqueous solution (1 mg ml^−1^, pH=7.5) and TiO_2_ nanoparticle (Svaya Nanotechnology, average diameter ≈20 nm) aqueous dispersion (0.03 wt%, pH=9.0). After depositing desired number of bilayers (30 or 45 bilayers) of PAH and TiO_2_, the substrates were calcined at 400 °C for 2 h to remove PAH from the surface. A scanning electron microscopy image shows that the surface is highly porous (see inset (i) in Fig. 1a).

### Fabrication of dye-sensitized TiO_2_ surfaces

Solutions (0.3 mM) of N3 dye (Sigma Aldrich), D149 dye (Sigma Aldrich) and Chlorin dye (Frontier Scientific) were prepared in anhydrous ethanol (Fischer Scientific). Note that N3 dye, D149 dye and Chlorin dye denote cis-bis(isothiocyanato) bis(2,2′-bipyridyl-4,4′-dicarboxylato ruthenium (II), 5-[[4-[4-(2,2-diphenylethenyl)phenyl]-1,2,3,3a,4,8b-hexahydrocyclopent[b]indol-7 yl]methylene]-2-(3-ethyl-4-oxo-2-thioxo-5-thiazolidinylidene)-4-oxo-3-thiazolidineacetic acid and 13-carboxy-17-(2-carboxyethyl)-15-carboxymethyl-17,18-trans-dihydro-3-vinyl-8-ethyl-2,7,12,18 tetramethylporphyrin, respectively. Small pieces of substrates with TiO_2_ surface were dip-coated in the desired solution for 12 h followed by thorough rinsing with ethanol to remove any residual dye molecules from the surface. The substrates were then dried with nitrogen gas.

### *In situ* contact angle measurements

A small volume (=4 μl) of ionic aqueous droplet was placed onto a dye-sensitized TiO_2_ surface submerged in dodecane. Visible light (MI 150, Edmund Optics) was irradiated from the top of the droplet. Note that the intensity of the light was constant (*I*=145 mW cm^−2^) in all measurements. The contact angle measurements were conducted using a Ramé–Hart 590-F1 goniometer.

### Fabrication of a patterned dye-sensitized TiO_2_ surface

A glass slide was masked in a channel (5 mm wide × 15 mm long) by Kapton polyimide adhesive tape (ULINE) by manual application. A TiO_2_ film was prepared on the unmasked region using LBL deposition followed by calcination as described above. Subsequently, the substrate was dip-coated in a Chlorin dye solution for 12 h to obtain a Chlorin dye-sensitized TiO_2_ channel. The channel was then masked by attaching a crosslinked polydimethylsiloxane (x-PDMS) film. The substrate was treated with heptadecafluoro-1,1,2,2-tetrahydrodecyl trichlorosilane (Gelest) by vapour phase deposition at 90 °C for 1 h to obtain hydrophobic background.

### Demulsification of a brine-in-dodecane emulsion

A brine-in-dodecane emulsion (30:70 v:v) was prepared by mixing water with 10 wt% NaCl and dodecane using a stir bar at 1,000 r.p.m. The concentration of Span80 surfactant was 0.1 wt% to dodecane phase. The emulsion is added onto a stainless steel mesh coated with a Chlorin dye-sensitized TiO_2_ film that is sandwiched between the two vertical glass tubes. After addition of emulsion, visible light is illuminated onto the mesh surface to induce coalescence of brine droplets.

### Data availability

The authors declare that the data supporting the findings of this study are available within the article and its Supplementary Information files.

## Additional information

**How to cite this article:** Kwon, G. *et al*. Visible light guided manipulation of liquid wettability on photoresponsive surfaces. *Nat. Commun.* **8**, 14968 doi: 10.1038/ncomms14968 (2017).

**Publisher's note:** Springer Nature remains neutral with regard to jurisdictional claims in published maps and institutional affiliations.

## Supplementary Material

Supplementary Information Supplementary Figures, Supplementary Tables, Supplementary Notes and Supplementary References

Supplementary Movie 1 This movie demonstrates the photo-induced anisotropic wetting of a NaCl droplet on a Chlorin dye-sensitized TiO_2_ surface. When focused white light shines on one edge of the droplet, the droplet spreads towards the illuminated edge while the other side is pinned.

Supplementary Movie 2 This movie demonstrates the demulsification of Span80-stabilized brine (10 wt% NaCl in water)-in-dodecane emulsion. The stainless steel mesh (pore size ≈ 2 μm) coated with Chlorin dye-sensitized TiO_2_ nanostructured film is sandwiched between the two vertical glass tubes. Brine droplets that contact the Chlorin dye-sensitized TiO_2_ mesh surface spread and coalesce with other droplets under white light illumination. Within minutes of illumination, the brine-in-dodecane emulsion demulsifies into brine-rich and dodecane-rich phases.
