# Supplementary material for: Visible light guided manipulation of liquid wettability on photoresponsive surfaces
Source: Nat Commun. 2017 Apr 25;8:14968. doi: 10.1038/ncomms14968 (PMC5413974; doi:10.1038/ncomms14968)
Supplement: Supplementary Information — Supplementary Figures, Supplementary Tables, Supplementary Notes and Supplementary References [file ncomms14968-s1.pdf]

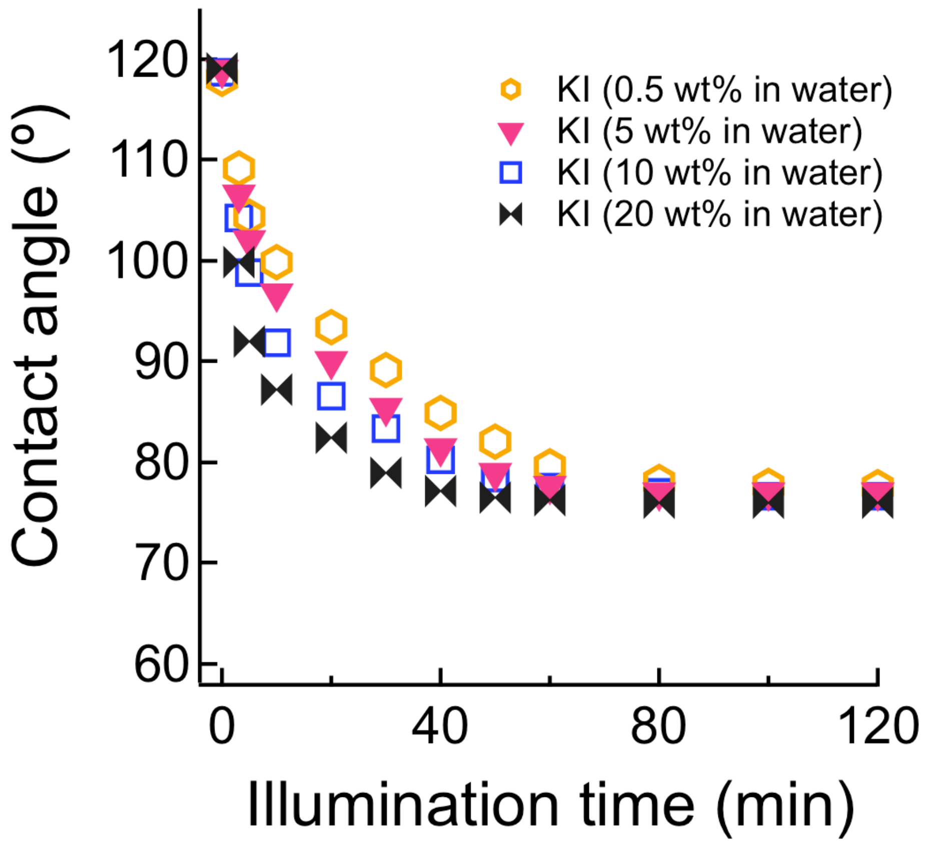

Supplementary Figure 1 | Evolution in the measured contact angles for potassium iodide (KI) droplets with various ionic concentrations on an N3 dye-sensitized TiO_2 surface as a function of illumination time. KI droplets with higher ionic concentration exhibit more rapid decrease in contact angles. The intensity of visible light illumination is 145 mW cm^{-2}.

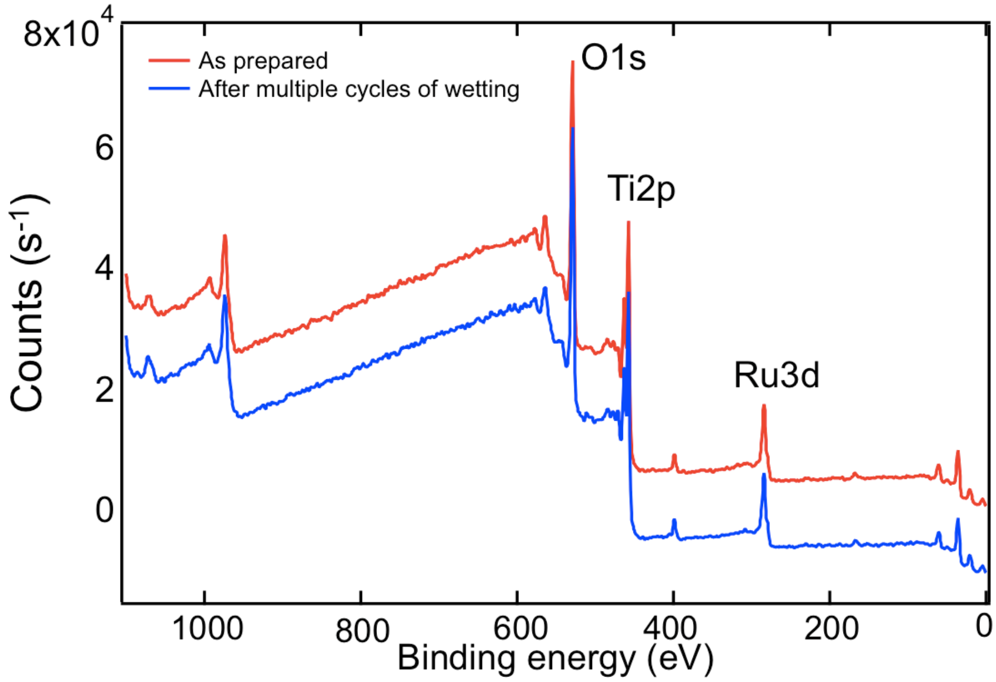

Supplementary Figure 2 | Survey XPS spectra of an N3 dye-sensitized TiO_2 surface before and after multiple wetting cycles under visible light illumination.

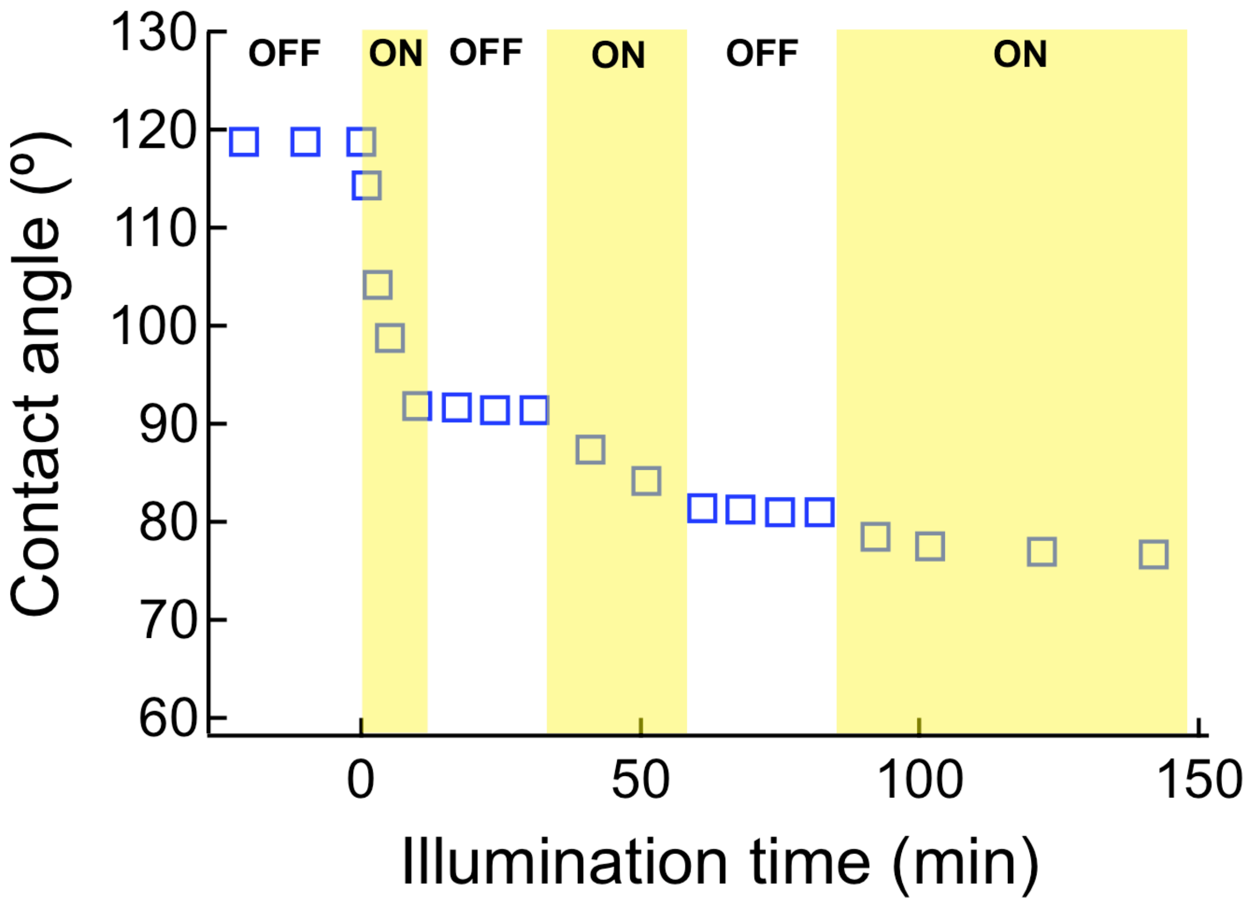

Supplementary Figure 3 | Evolution in the measured contact angles for a KI droplet (10 wt% in water) on an N3 dye-sensitized TiO_2 surface upon intermittent visible light illumination (Intensity = 145 mW cm^{-2}).

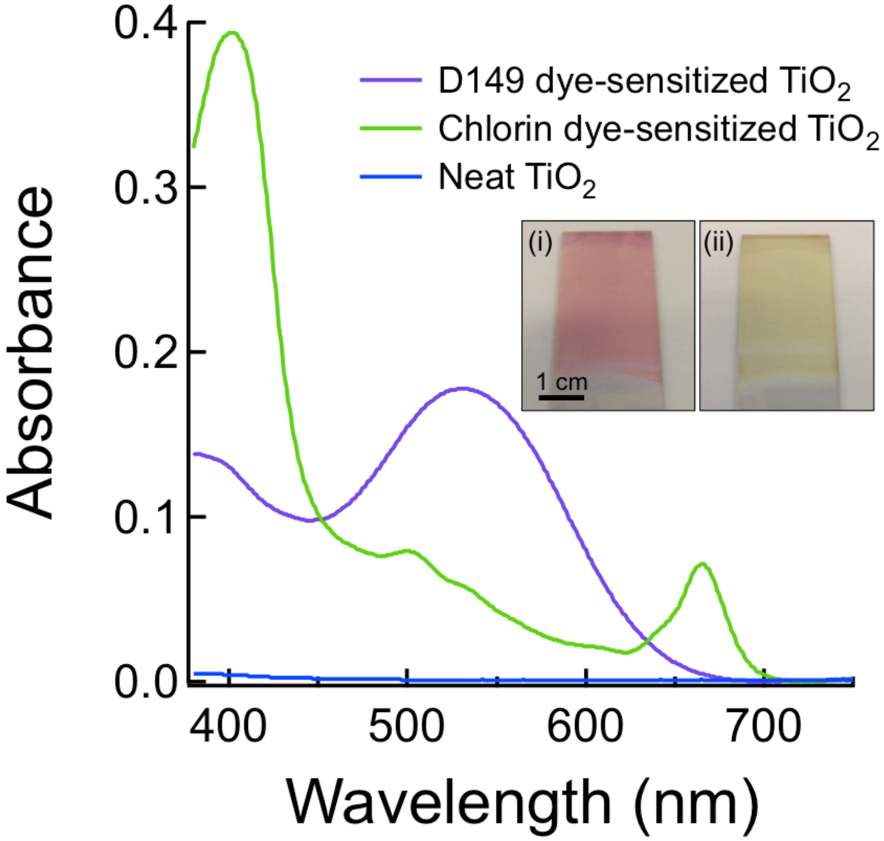

Supplementary Figure 4 | Visible light absorption data of a D149 dye-sensitized TiO_2 surface and a Chlorin dye-sensitized TiO_2 surface. Corresponding absorption data of an unsensitized neat TiO_2 surface is also shown for comparison. Insets: (i) A photograph of a D149 dye-sensitized TiO_2 surface and (ii) a photograph of a Chlorin dye-sensitized TiO_2 surface.

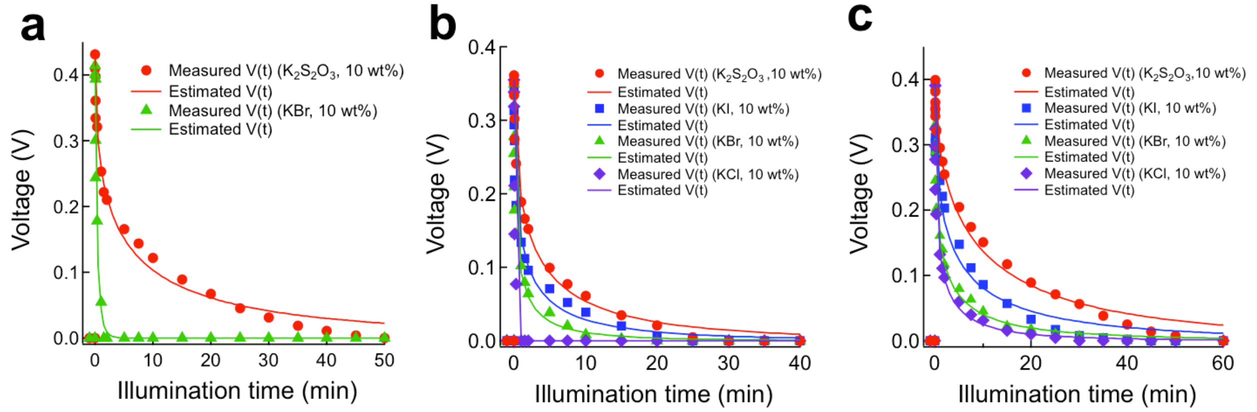

Supplementary Figure 5 | Measured voltages across the contacting liquids and the underlying dye-sensitized TiO_2 surfaces. (a) A plot of measured voltages across the contacting liquids ($K_2S_2O_3$ and KBr) on the N3 dye-sensitized TiO_2 surface under visible light illumination. **(b,c)** Plots of measured voltages across the contacting liquids ($K_2S_2O_3$, KI, KBr and KCl) on the D149 dye-sensitized TiO_2 surface and Chlorin dye-sensitized TiO_2 surface, respectively, under visible light illumination. The intensity of the visible light illumination is 145 mW cm^{-2} for all measurements.

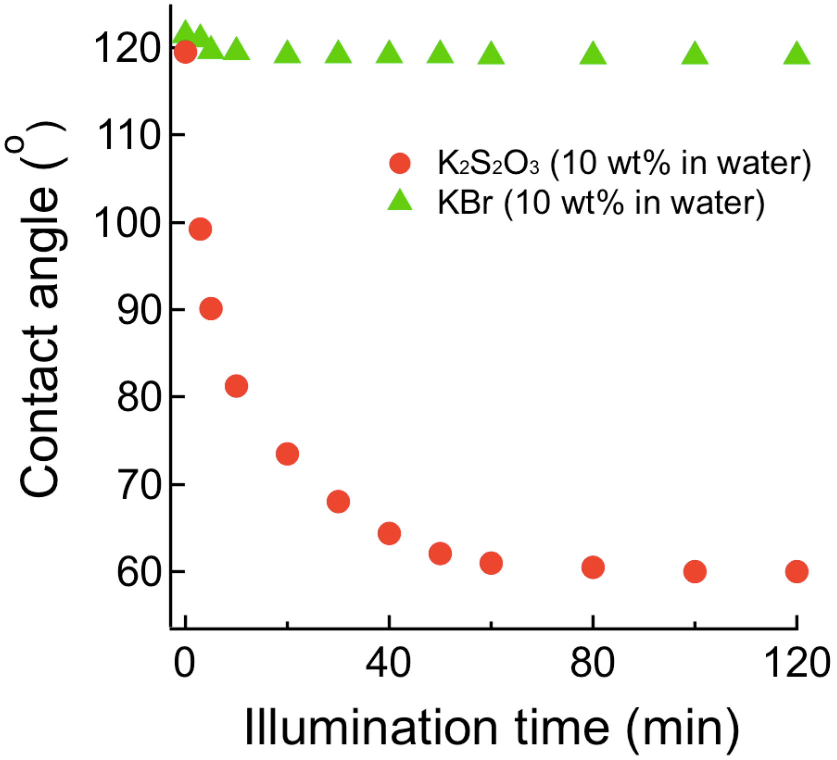

Supplementary Figure 6 | Evolution in the measured contact angles for potassium thiosulfate ($K_2S_2O_3$) and potassium bromide (KBr) droplets on an N3 dye-sensitized TiO_2 surface upon visible light illumination. The contact angles for $K_2S_2O_3$ decrease with increasing illumination time while those for KBr remain almost constant. The intensity of visible light is 145 mW cm^{-2}.

Supplementary Table 1 | Measured contact angles for potassium chloride (KCl) droplets with various concentrations on an N3 dye-sensitized TiO_2 surface before and after illumination for 120 min.

	0.5 wt% KCl	5 wt% KCl	10 wt% KCl	20 wt% KCl
θ^*_{KCl} (t = 0)	120.6°	120.9°	121.2°	121.6°
θ^*_{KCl} (t = 120 min)	119.2°	119.1°	119.9°	119.2°
$\Delta\theta^*(=\theta^*_{t=0}-\theta^*_{t=120\,\mathrm{min}})$	1.4°	1.8°	1.3°	2.4°

Supplementary Table 2 | The values of τ_d and α found in the voltage predictions using our fractional RC circuit model.

		τ_d (sec)	α
N3 dye-sensitized TiO_2	$K_2S_2O_3$	271±43	0.45±0.04
	KI	130±24	0.42±0.04
	KBr	20.6±9.4	0.76±0.28
	KCl	11.5±4.8	0.99±0.43
D149 dye-sensitized TiO_2	$K_2S_2O_3$	156.9±25	0.48±0.05
	KI	75±19.7	0.44±0.06
	KBr	29.9±10.8	0.41±0.08
	KCl	8.4±4.3	0.98±0.53
Chlorin dye-sensitized TiO_2	$K_2S_2O_3$	530±67	0.54±0.05
	KI	216±55	0.45±0.07
	KBr	76.6±16.8	0.4±0.04
	KCl	50.7±11.6	0.41±0.05

Supplementary Note 1. Estimation of the ratio of total surface area per unit projected area.

To qualify the porosity of the nanoporous TiO_2 film we calculate the ratio of the total internal surface area of the nanoporous structure per unit projected area. The absorbance of N3 dye-sensitized TiO_2 surface at 478 nm is 0.1736 (see Fig. 1a in the main text). The extinction coefficient of N3 dye at 478 nm is $e_{478nm} = 1.88 \times 10^7 cm^2 mol^{-1}$ [1]. The dye concentration on the surface can be calculated by dividing the absorbance with the extinction coefficient. This yields $9.23 \times 10^{-9} mol\, cm^{-2}$. Considering that each dye molecule occupies an area of ≈ 1 nm^2 [1], the internal surface area is estimated to be 56 cm^2 for each 1 cm^2 projected area.

Supplementary Note 2. Measured contact angles for KI and KCl droplets with various concentrations on an N3 dye-sensitized TiO_2 surface under visible light illumination.

We measured the advancing contact angles for aqueous KI and KCl droplets with a range of different concentrations on an N3 dye-sensitized TiO_2 surface upon visible light illumination. Supplementary Figure 1 shows the contact angles for aqueous KI droplets with 0.5 wt%, 5 wt%, 10 wt% and 20 wt% KI as a function of illumination time. It shows that KI droplets with higher ionic concentration exhibit a more rapid decrease in the contact angles under optical illumination. On the other hand, the contact angles for KCl droplets remained almost unchanged during illumination and this negligible change in the contact angles remained invariant of the ionic concentration (see Supplementary Table 1).

Supplementary Note 3. X-ray photoelectron spectroscopy (XPS) analysis of an N3 dye-sensitized TiO_2 surface after visible light illumination.

To verify that the surface chemistry of our N3 dye-sensitized TiO_2 surface remains unaffected after visible light illumination, XPS measurements were conducted using a PHI 5600 ESCA multi-detection system with a base pressure of 1×10^{-10} Torr. The X-ray radiation was the monochromatic Al Kα line (1486.7 eV); the X-ray spot size and the take-off angle were 0.8 mm and 45°, respectively.

The survey spectra of the N3 dye-sensitized TiO_2 before and after multiple cycles of wetting under visible light illumination are shown in Supplementary Figure 2. The photoelectron peaks in the spectra can be attributed to titanium (Ti), oxygen (O) and ruthenium (Ru). The origin of ruthenium is due to N3 dye molecules adsorbed on the TiO_2 surface. The surface compositions were obtained by normalizing the area under the curve. We found that the atomic concentration ratio of titanium (Ti2p) and ruthenium (Ru3d) of our N3 dye-sensitized TiO_2 surface remained unchanged (72.1:27.9, atomic%:atomic%) even after multiple cycles of wetting under visible light illumination. This indicates that our N3 dye-sensitized TiO_2 surface is robust against through optical illumination.

Supplementary Note 4. Prediction of voltages between the contacting liquid droplets and our N3 dye-sensitized TiO_2 surfaces.

As discussed in the main text, the electrolytic double layer formed at the liquid-solid interface under incident illumination can be considered as a capacitor. We predict the evolution in the time-dependent voltages by assuming that the capacitor is discharged over time. When a perfect capacitor is discharged in an RC circuit, the voltage decays exponentially over time and the decay is characterized by a voltage response of the form $V(t) \propto \exp[-(t/\tau_d)]$, where τ_d is the relaxation time constant. However our electrolytic double layer does not form a perfect capacitor. Our dye-sensitized TiO_2 surface possesses nanometric pores contacting oil (dodecane) phase which leads also to some resistive characteristic. When such an 'imperfect' capacitor is discharged, the voltage can be characterized by a stretched exponential function, $V(t) \propto \exp[-(t/\tau_d)^{\alpha}]$, where α ($0 < \alpha \leq 1$) is the fractional derivative order[2] (see also Eqn. (1) in main text). When α =1, the stretched exponential reduces to the simple exponential. We utilized Eqn. (1) to fit the evolution in voltages between the KI (or KCl) droplet and the surface. We found that our measured voltages match well with Eqn. (1) with τ_d = 130 sec and α = 0.42 for KI droplet and τ_d = 11.5 sec and α = 0.988 for the KCl droplet, respectively. See Supplementary Note 7 for a comparison of the surface response characteristics for different dyes and ionic concentrations.

Supplementary Note 5. Measured contact angles for a KI droplet on an N3 dye-sensitized TiO_2 surface upon intermittent visible light illumination.

We measured the contact angles for a KI droplet (10 wt% in water) on an N3 dye-sensitized TiO_2 surface upon intermittent visible light illumination. Supplementary Figure 3 shows the contact angles for a KI droplet as a function of illumination time. The contact angle decreases progressively from $\theta^*_{KI,t=0}$ = 119° after the onset of illumination and reaches $\theta^*_{KI,t=10\,\mathrm{min}}$ = 92° at t = 10 min. When the illumination is turned off at t = 10 min, the evolution in the contact angle halts instantaneously and the contact angle remains unchanged. When the illumination is turned on at t = 30 min, we observed that the contact angle starts to decrease again. The illumination is again turned off at t = 60 min and the contact angle is found to be almost constant ($\theta^*_{KI,t=60\,\mathrm{min}} \approx \theta^*_{KI,t=90\,\mathrm{min}} \approx$ 81°) in the absence of optical illumination. The contact angle starts to decrease after the onset of illumination at t = 90 min and finally approaches $\theta^*_{KI,t=160\,\mathrm{min}}$ = 76°.

Supplementary Note 6. Visible light absorbance data of a D149 dye-sensitized TiO_2 surface and a Chlorin dye-sensitized TiO_2 surface.

We fabricated two different dye-sensitized TiO_2 surfaces utilizing D149 dye and Chlorin dye (see Methods). Adsorption of D149 dye molecules leads to a light-red coloration of the surface (see inset (i) in Supplementary Figure 4). A Chlroin dye sensitized TiO_2 surface exhibits a light-green color (see inset (ii) in Supplementary Figure 4). Supplementary Figure 4 shows the absorption spectra of a D149 dye-sensitized TiO_2 surface and a Chlorin dye-sensitized TiO_2 surface. It is evident that both surfaces absorb a broad range of lights in the visible spectrum (390 nm ≤ λ ≤ 700 nm).

Supplementary Note 7. Measured voltages between the contacting liquid droplets and dye-sensitized TiO_2 surfaces.

We measured the *in situ* voltages established across the contacting liquids and our dye-sensitized TiO_2 surfaces. Here we utilized four different ionic aqueous droplets ($K_2S_2O_3$, KI, KBr and KCl, all concentrations are 10 wt% in water) as probe contacting liquids.

(i) N3 dye-sensitized TiO_2 surface: Supplementary Figure 5a shows the measured voltages across the contacting liquids ($K_2S_2O_3$ and KBr) and the N3 dye-sensitized TiO_2 surface while illuminating with visible light. Immediately after the onset of illumination, a potential difference ($V_{K_2S_2O_3,\ t=0} = 0.43$ V and $V_{KBr,\ t=0} = 0.41$ V) is observed. While the voltage gradually decreases between the $K_2S_2O_3$ droplet and the surface over time, we observed a rapid decrease in the voltage between the KBr droplet and the surface. This is because the bromide ion (Br^-) cannot effectively reduce the oxidized N3 dye as its reduction potential is higher (more positive) than the HOMO energy level of the N3 dye (see main text). The evolution in our measured voltages between the $K_2S_2O_3$ droplet and ITO match well with Eqn. (1) in the main text (see Supplementary Note 4) with τ_d = 271 sec and α = 0.45 (see Supplementary Figure 5a and Supplementary Table 2). We also found that the measured voltages between the KBr droplet and the surface can be well described with τ_d = 20.6 sec and α = 0.76 (see Supplementary Figure 5a).

(ii) D149 dye-sensitized TiO_2 surface: Supplementary Figure 5b shows the measured *in situ* voltages between the contacting liquids and the D149 dye-sensitized TiO_2 surface. After a potential difference ($V_{t=0} \approx 0.355$ V) is established for all contacting liquids upon the onset of optical illumination, a gradual decrease in the voltage is observed for $K_2S_2O_3$, KI and KBr. It is noteworthy that the KBr droplet exhibited a prolonged voltage difference on a D149 dye-sensitized TiO_2 surface in contrast to a rapid decrease on an N3 dye-sensitized TiO_2 surface (see Supplementary Figure 5a). This is because the HOMO energy level of a D149 dye is lower (less positive) than the reduction potential of bromide which allows the regeneration process of oxidized D149 dye[3] (see main text). In contrast, the measured voltage between the KCl droplet and the surface decreases rapidly and reaches zero within a minute of illumination. As expected, this is because the reduction potential of chloride is higher (more positive) than the HOMO energy level of D149 dye which hinders effective regeneration of oxidized dye (see main text). Eqn. (1) in the main text describes the measured voltages well with various values of τ_d and α (see Supplementary Table 2).

(iii) Chlorin dye-sensitized TiO_2 surface: Supplementary Figure 5c shows the measured *in situ* voltages across the contacting liquids and the Chlorin dye-sensitized TiO_2 surface. As the HOMO energy level of a Chlorin dye is higher (more positive) than the reduction potential of all contacting liquids ($K_2S_2O_3$, KI, KBr and KCl), we observed that voltages for all contacting liquid droplets decrease gradually with increasing illumination time. This leads to spreading of all

contacting liquids including KCl droplets (see Fig. 3c in the main text).

Supplementary Table 2 lists the values of τ_d and α found in the voltage predictions using our fractional RC circuit model.

Supplementary Note 8. Measured contact angles for $K_2S_2O_3$ and KBr droplets on an N3 dye-sensitized TiO_2 surface under visible light illumination.

We also measured the *in situ* contact angles for two liquid droplets: $K_2S_2O_3$ and KBr (10 wt% in water) on an N3 dye-sensitized TiO_2 surface. Supplementary Figure 6 shows the evolution in the macroscopic contact angles for $K_2S_2O_3$ and KBr droplets as a function of illumination time. The contact angles for the $K_2S_2O_3$ droplet decrease from $\theta^*_{t=0}$ = 119° with increasing illumination time before it approaches $\theta^*_{t=120\,\mathrm{min}}$ = 60° while those for KBr remain almost constant during illumination ($\Delta\theta^* \approx 2°$ where $\Delta\theta^* = \theta^*_{t=0} - \theta^*_{t=120\,\mathrm{min}}$).

Supplementary References

1. Nazeeruddin, M. K., Liska, P., Moser, J., Vlachopoulos, N. & Grätzel, M., *Helv. chim. Acta* **73**, 1788−1803 (1990).
2. Radwan, A. G. & Salama, K. N. Fractional-Order RC and RL Circuits. *Circ Syst Signal Pr* **31**, 1901-1915 (2012).
3. Wang, Z-S., Sayama, K. & Sugihara, H., Efficient Eosin Y Dye-Sensitized Solar Cell Containing Br^-/Br^{3-} Electrolyte. *J Phys Chem B* **109**, 22449-22455 (2005).
